# Supplementary material for: Elucidation of trophic interactions in an unusual single-cell nitrogen-fixing symbiosis using metabolic modeling
Source: PLoS Comput Biol. 2021 May 7;17(5):e1008983. doi: 10.1371/journal.pcbi.1008983 (PMC8143392; doi:10.1371/journal.pcbi.1008983)
Supplement: S1 Text — (DOCX) [file pcbi.1008983.s005.docx]

**Supplementary Material**

1. *Genome Scale Model Reconstruction*

**Table 1: Statistics for host and UCYN-A genome-scale models.**

|  | **Host** | **UCYN-A** |
| --- | --- | --- |
| **Metabolites** | 3,183 | 1,324 |
| **Genes** | 714 | 401 |
| **Reactions** | 3,109 | 1,330 |
| **Enzymatic reactions** | 1,700 | 590 |
| **Gapfilled reactions** | 18 | 22 |
| **with GPR assigned** | 1,663 | 590 |
| **Transport reactions** | 331 | 70 |
| **Transport reactions  with GPR assigned** | 53 | 18 |

1. *Impact of fixed nitrogen availability on the host-UCYN-A symbiosis*

The host nitrogen uptake was varied from zero (i.e., relying solely on UCYN-A) to the amount needed for maximal growth (i.e., all nitrogen requirements being satisfied from the environment). Carbon and nitrogen sharing between the two partners was also allowed to vary from the experimentally observed ratios [1]. The resultant maximal host biomass production flux and UCYN-A N_2_ fixation flux was then calculated for each case (Figure 1). As expected, the host biomass increases as the nitrogen supply is increased (either from UCYN-A or the environment), but also competes with carbon transfer to UCYN-A. In the limiting case when UCYN-A is the sole source of fixed nitrogen, increasing carbon transfer translates to a higher nitrogen fixation flux (due to increased availability of reducing equivalents) but only to the extent wherein it does not compete with carbon sequestration for biomass production. At the opposite end of the spectrum, where all nitrogen requirements are fulfilled by the environment, increasing carbon transfer leads to a decrease in host biomass production. This transition from nitrogen-limited to carbon-limited growth thus dictates the limits of this symbiotic system.


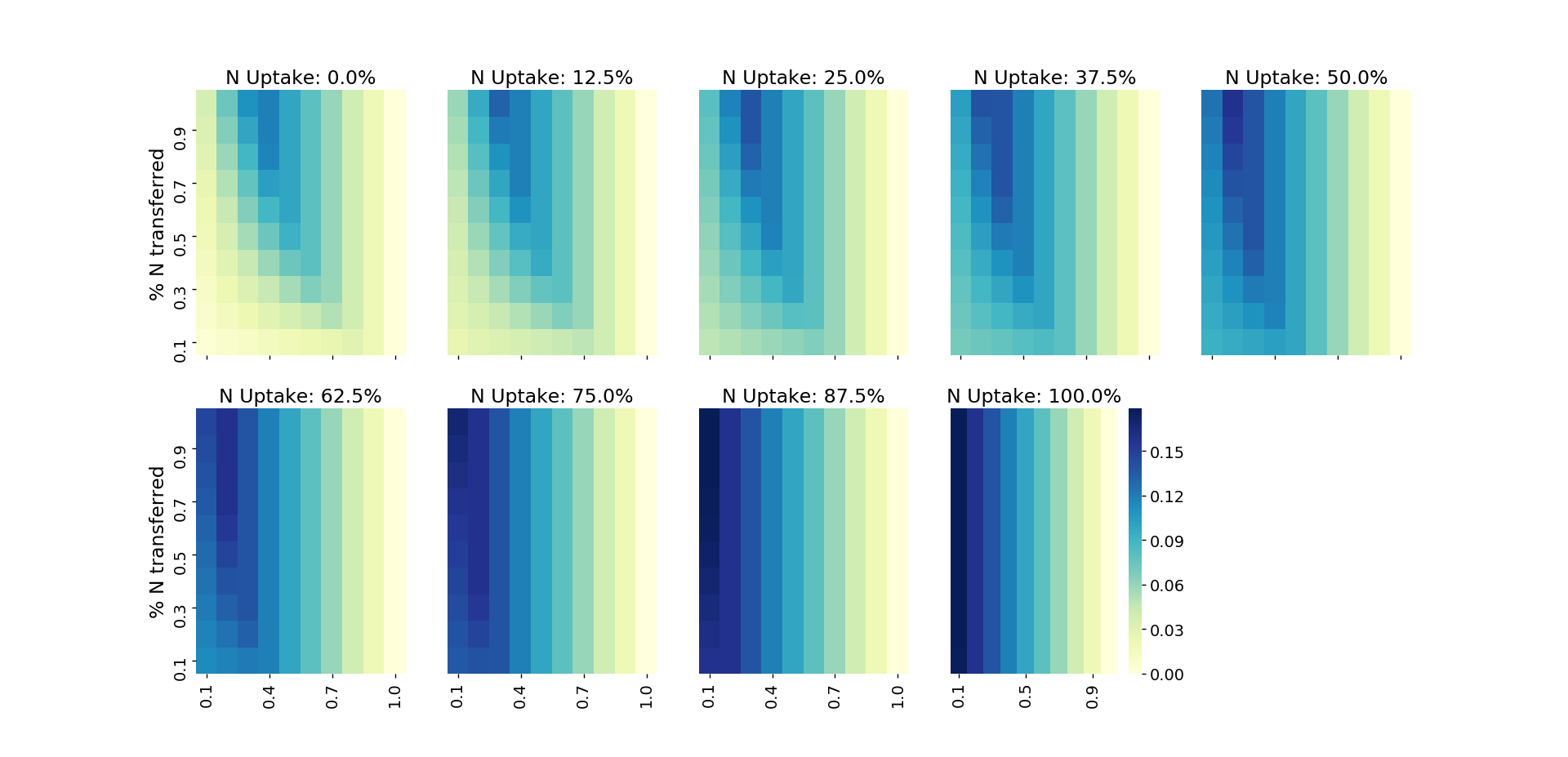


**Figure 1: Predicted algal host growth rate with variations in metabolite transfer and host nitrogen uptake.** Nitrogen transfer (from UCYN-A to host, y-axis) and carbon transfer (from host to UCYN-A, x-axis) were varied, and the maximal host biomass production flux calculated (in hr^-1^, see color bar on bottom right). Nitrogen uptake for the host was also varied, ranging from zero (denoting no nitrogen supplementation from the environment and relying solely on UCYN-A) to 100% (denoting all nitrogen requirements being satisfied from the environment) (see individual plot titles).

1. Metabolic Control Analysis in UCYN-A

A metabolic control analysis [2], [3] was performed to analyze the effect of reaction-level flux perturbations in UCYN-A on the maximal N_2_ fixation and host biomass production flux. Host biomass production was first maximized, and then the maximal N_2_fixation flux calculated at that value, followed by parsimonious flux balance analysis to determine the reference flux distribution.

Flux through every UCYN-A metabolic reaction was then perturbed (by decreasing it by 10% of the reference value), and the resultant host biomass production and UCYN-A N_2_ fixation determined. Flux control coefficients (FCC) for host biomass (${FCC}_{host biomass, j}$) and UCYN-A N_2_ fixation (${FCC}_{N_{2} fixation,j}$) for every UCYN-A metabolic reaction (i.e., $j \in\boldsymbol{J}_{\boldsymbol{UCYN-A}}$) were calculated as –

$${FCC}_{host biomass, j}= \frac{\frac{\Delta v_{biomass}}{v_{biomass, ref}}}{\frac{\Delta v_{j}}{v_{j, ref}}}, \forall j \in\boldsymbol{J}_{\boldsymbol{UCYN-A}}$$

$${FCC}_{N_{2} fixation, j}= \frac{\frac{\Delta v_{N_{2} fixation}}{v_{N_{2} fixation, ref}}}{\frac{\Delta v_{j}}{v_{j, ref}}}, \forall j \in\boldsymbol{J}_{\boldsymbol{UCYN-A}}$$

Where $v_{biomass, ref}$, $v_{N_{2} fixation, ref}$, and $v_{j, ref}$ refer to the host biomass, UCYN-A N_2_ fixation, and flux for a reaction $j$ in the reference flux distribution, respectively. $\Delta v_{j}$ refers to the imposed flux perturbation for reaction $j$, and $\Delta v_{biomass}$ and $\Delta v_{N_{2} fixation}$ are the corresponding changes in host biomass and UCYN-A nitrogen fixation flux.

As expected, reactions involved in producing reductants NAD(P)H and ATP had a high impact on both host biomass and N2 fixation (such as PSI, uptake hydrogenase, and cyclic electron flow) (see Table 2 below). Reactions involved in riboflavin metabolism and GTP synthesis (which serves as the precursor for riboflavin) also affected host biomass production, which is likely to change if external sources of riboflavin are available to the host.

**Table 2. List of UCYN-A metabolic reactions with a non-zero flux control coefficient for host biomass synthesis and/or N_2_ fixation.**

+ Note: simulations were carried out without riboflavin supplementation in the growth media, and thus MCA FCC values are likely to change when external riboflavin is available to the host.

1. *UCYN-A Gene and Reaction Essentiality*

An essentiality study was performed by systematically deleting UCYN-A model reactions and determining the subsequent maximal production flux for each biomass precursor. This analysis indicates that 154 reactions (or 25.4%) are essential in UCYN-A, which translates to 155 essential metabolic genes (or 38.8%). This fraction is similar to other minimal cyanobacteria (such as *P. marinus* with 47% essential genes (Casey et al., mSys, 2016)), reinforcing the streamlined metabolism from adaptive gene loss hypothesis (Bombar et al., ISME Jour., 2014).

**Table 3. List of essential reactions, the corresponding metabolic pathway, and genes in UCYN-A, alongside the number and metabolite name of biomass precursor(s) that are blocked upon its deletion.**

| **Essential Metabolic Reactions** | | | **Blocked Biomass Precursor(s)** | |
| --- | --- | --- | --- | --- |
| **Reaction ID** | **Pathway** | **Associated Gene(s)** | **Number** | **Metabolite(s)** |
| rxn02974 | Amino and Nucleotide Sugar Metabolism | UCYN_02170 | 1 | cdp-3,6-dideoxy-d-mannose |
| rxn02432 | Amino and Nucleotide Sugar Metabolism | UCYN_00940 | 1 | cdp-3,6-dideoxy-d-mannose |
| rxn00702 | Amino and Nucleotide Sugar Metabolism | UCYN_11560 | 1 | cdp-3,6-dideoxy-d-mannose |
| rxn01675 | Amino and Nucleotide Sugar Metabolism | UCYN_08010, UCYN_02150 | 1 | dTDP-rhamnose |
| rxn00293 | Amino and Nucleotide Sugar Metabolism | UCYN_00650 | 1 | lipid A disaccharide |
| rxn01485 | Amino and Nucleotide Sugar Metabolism | UCYN_10200 | 1 | lipid A disaccharide |
| rxn03638 | Amino and Nucleotide Sugar Metabolism | UCYN_00650 | 1 | lipid A disaccharide |
| rxn01750 | Amino and Nucleotide Sugar Metabolism | UCYN_08070 | 1 | cdp-3,6-dideoxy-d-mannose |
| rxn04027 | Amino and Nucleotide Sugar Metabolism | UCYN_02170 | 7 | sulfoquinovosyldiacylglycerol n-c14:0, sulfoquinovosyldiacylglycerol n-c16:0, sulfoquinovosyldiacylglycerol n-c16:1, sulfoquinovosyldiacylglycerol n-c18:0, sulfoquinovosyldiacylglycerol n-c18:1, sulfoquinovosyldiacylglycerol n-c18:2, sulfoquinovosyldiacylglycerol n-c18:3 |
| rxn00214 | Amino and Nucleotide Sugar Metabolism | UCYN_08800 | 14 | digalactosyl-diacylglycerol (n-c14), digalactosyl-diacylglycerol (n-c16), digalactosyl-diacylglycerol (n-c16:1), digalactosyl-diacylglycerol (n-c18:0), digalactosyl-diacylglycerol (n-c18:1), digalactosyl-diacylglycerol (n-c18:2), Digalactosyl-diacylglycerol(n-C18:3), 1,2-diacyl-3-beta-d-galactosyl-sn-glycerol (n-c14), 1,2-diacyl-3-beta-d-galactosyl-sn-glycerol (n-c16), 1,2-diacyl-3-beta-d-galactosyl-sn-glycerol (n-c16:1), 1,2-diacyl-3-beta-d-galactosyl-sn-glycerol (n-c18:0), 1,2-diacyl-3-beta-d-galactosyl-sn-glycerol (n-c18:1), 1,2-diacyl-3-beta-d-galactosyl-sn-glycerol (n-c18:2), 1,2-diacyl-3-beta-d-galactosyl-sn-glycerol (n-c18:3) |
| rxn00213 | Amino and Nucleotide Sugar Metabolism | UCYN_11560 | 22 | sulfoquinovosyldiacylglycerol n-c14:0, sulfoquinovosyldiacylglycerol n-c16:0, sulfoquinovosyldiacylglycerol n-c16:1, sulfoquinovosyldiacylglycerol n-c18:0, sulfoquinovosyldiacylglycerol n-c18:1, sulfoquinovosyldiacylglycerol n-c18:2, sulfoquinovosyldiacylglycerol n-c18:3, udp-glucose, digalactosyl-diacylglycerol (n-c14), digalactosyl-diacylglycerol (n-c16), digalactosyl-diacylglycerol (n-c16:1), digalactosyl-diacylglycerol (n-c18:0), digalactosyl-diacylglycerol (n-c18:1), digalactosyl-diacylglycerol (n-c18:2), Digalactosyl-diacylglycerol(n-C18:3), 1,2-diacyl-3-beta-d-galactosyl-sn-glycerol (n-c14), 1,2-diacyl-3-beta-d-galactosyl-sn-glycerol (n-c16), 1,2-diacyl-3-beta-d-galactosyl-sn-glycerol (n-c16:1), 1,2-diacyl-3-beta-d-galactosyl-sn-glycerol (n-c18:0), 1,2-diacyl-3-beta-d-galactosyl-sn-glycerol (n-c18:1), 1,2-diacyl-3-beta-d-galactosyl-sn-glycerol (n-c18:2), 1,2-diacyl-3-beta-d-galactosyl-sn-glycerol (n-c18:3) |
| rxn01489 | Carotenoid Biosynthesis | UCYN_01090 | 1 | all-trans-zeta-carotene |
| rxn05031 | Carotenoid Biosynthesis | UCYN_01090 | 1 | all-trans-zeta-carotene |
| rxn00126 | Cysteine and Methionine Metabolism | UCYN_06940 | 1 | s-adenosyl-l-methionine |
| rxn05327 | Fatty Acid Synthesis | UCYN_08930 | 1 | lipid A disaccharide |
| rxn05324 | Fatty Acid Synthesis | UCYN_08930 | 1 | lipid A disaccharide |
| rxn05325 | Fatty Acid Synthesis | UCYN_08930 | 1 | lipid A disaccharide |
| rxn05331 | Fatty Acid Synthesis | UCYN_10780 | 1 | lipid A disaccharide |
| rxn05333 | Fatty Acid Synthesis | UCYN_10780 | 1 | lipid A disaccharide |
| rxn05338 | Fatty Acid Synthesis | UCYN_08150 | 1 | lipid A disaccharide |
| rxn05348 | Fatty Acid Synthesis | UCYN_11720 | 1 | lipid A disaccharide |
| rxn05345 | Fatty Acid Synthesis | UCYN_11720 | 1 | lipid A disaccharide |
| rxn05340 | Fatty Acid Synthesis | UCYN_08150 | 1 | lipid A disaccharide |
| rxn05342 | Fatty Acid Synthesis | UCYN_08150 | 1 | lipid A disaccharide |
| rxn05343 | Fatty Acid Synthesis | UCYN_11720 | 1 | lipid A disaccharide |
| DESAT183_ACP | Fatty Acid Synthesis | UCYN_05530 | 3 | sulfoquinovosyldiacylglycerol n-c18:3, Digalactosyl-diacylglycerol(n-C18:3), 1,2-diacyl-3-beta-d-galactosyl-sn-glycerol (n-c18:3) |
| DESAT161_ACP | Fatty Acid Synthesis | UCYN_05530 | 4 | sulfoquinovosyldiacylglycerol n-c16:1, phosphatidylglycerol dihexadec-9-enoyl, digalactosyl-diacylglycerol (n-c16:1),  1,2-diacyl-3-beta-d-galactosyl-sn-glycerol (n-c16:1) |
| DESAT182_ACP | Fatty Acid Synthesis | UCYN_05530 | 6 | sulfoquinovosyldiacylglycerol n-c18:2, sulfoquinovosyldiacylglycerol n-c18:3, digalactosyl-diacylglycerol (n-c18:2), Digalactosyl-diacylglycerol(n-C18:3), 1,2-diacyl-3-beta-d-galactosyl-sn-glycerol (n-c18:2), 1,2-diacyl-3-beta-d-galactosyl-sn-glycerol (n-c18:3) |
| DESAT181_ACP | Fatty Acid Synthesis | UCYN_05530 | 10 | sulfoquinovosyldiacylglycerol n-c18:1, sulfoquinovosyldiacylglycerol n-c18:2, sulfoquinovosyldiacylglycerol n-c18:3, phosphatidylglycerol dioctadec-11-enoyl, digalactosyl-diacylglycerol (n-c18:1), digalactosyl-diacylglycerol (n-c18:2), Digalactosyl-diacylglycerol(n-C18:3), 1,2-diacyl-3-beta-d-galactosyl-sn-glycerol (n-c18:1), 1,2-diacyl-3-beta-d-galactosyl-sn-glycerol (n-c18:2), 1,2-diacyl-3-beta-d-galactosyl-sn-glycerol (n-c18:3) |
| rxn07577 | Fatty Acid Synthesis | UCYN_08150 | 14 | sulfoquinovosyldiacylglycerol n-c18:0, sulfoquinovosyldiacylglycerol n-c18:1, sulfoquinovosyldiacylglycerol n-c18:2,  sulfoquinovosyldiacylglycerol n-c18:3, phosphatidylglycerol dioctadecanoyl, phosphatidylglycerol dioctadec-11-enoyl, digalactosyl-diacylglycerol (n-c18:0), digalactosyl-diacylglycerol (n-c18:1), digalactosyl-diacylglycerol (n-c18:2), Digalactosyl-diacylglycerol(n-C18:3), 1,2-diacyl-3-beta-d-galactosyl-sn-glycerol (n-c18:0), 1,2-diacyl-3-beta-d-galactosyl-sn-glycerol (n-c18:1), 1,2-diacyl-3-beta-d-galactosyl-sn-glycerol (n-c18:2), 1,2-diacyl-3-beta-d-galactosyl-sn-glycerol (n-c18:3) |
| rxn07576 | Fatty Acid Synthesis | UCYN_11720 | 14 |  |
| rxn07579 | Fatty Acid Synthesis | UCYN_08930 | 14 |  |
| rxn07578 | Fatty Acid Synthesis | UCYN_10780 | 14 |  |
| rxn05328 | Fatty Acid Synthesis | UCYN_08930 | 22 | sulfoquinovosyldiacylglycerol n-c16:0, sulfoquinovosyldiacylglycerol n-c16:1, sulfoquinovosyldiacylglycerol n-c18:0, sulfoquinovosyldiacylglycerol n-c18:1, sulfoquinovosyldiacylglycerol n-c18:2, sulfoquinovosyldiacylglycerol n-c18:3, phosphatidylglycerol dihexadecanoyl, phosphatidylglycerol dihexadec-9-enoyl, phosphatidylglycerol dioctadecanoyl, phosphatidylglycerol dioctadec-11-enoyl, digalactosyl-diacylglycerol (n-c16), digalactosyl-diacylglycerol (n-c16:1), digalactosyl-diacylglycerol (n-c18:0), digalactosyl-diacylglycerol (n-c18:1), digalactosyl-diacylglycerol (n-c18:2), Digalactosyl-diacylglycerol(n-C18:3), 1,2-diacyl-3-beta-d-galactosyl-sn-glycerol (n-c16), 1,2-diacyl-3-beta-d-galactosyl-sn-glycerol (n-c16:1), 1,2-diacyl-3-beta-d-galactosyl-sn-glycerol (n-c18:0), 1,2-diacyl-3-beta-d-galactosyl-sn-glycerol (n-c18:1), 1,2-diacyl-3-beta-d-galactosyl-sn-glycerol (n-c18:2), 1,2-diacyl-3-beta-d-galactosyl-sn-glycerol (n-c18:3) |
| rxn05332 | Fatty Acid Synthesis | UCYN_10780 | 22 |  |
| rxn05336 | Fatty Acid Synthesis | UCYN_08150 | 22 |  |
| rxn05344 | Fatty Acid Synthesis | UCYN_11720 | 22 |  |
| rxn05465 | Fatty Acid Synthesis | UCYN_01150 | 23 | sulfoquinovosyldiacylglycerol n-c16:0, sulfoquinovosyldiacylglycerol n-c16:1, sulfoquinovosyldiacylglycerol n-c18:0, sulfoquinovosyldiacylglycerol n-c18:1, sulfoquinovosyldiacylglycerol n-c18:2, sulfoquinovosyldiacylglycerol n-c18:3, lipid A disaccharide, phosphatidylglycerol dihexadecanoyl, phosphatidylglycerol dihexadec-9-enoyl, phosphatidylglycerol dioctadecanoyl, phosphatidylglycerol dioctadec-11-enoyl, digalactosyl-diacylglycerol (n-c16), digalactosyl-diacylglycerol (n-c16:1), digalactosyl-diacylglycerol (n-c18:0), digalactosyl-diacylglycerol (n-c18:1), digalactosyl-diacylglycerol (n-c18:2), Digalactosyl-diacylglycerol(n-C18:3), 1,2-diacyl-3-beta-d-galactosyl-sn-glycerol (n-c16), 1,2-diacyl-3-beta-d-galactosyl-sn-glycerol (n-c16:1), 1,2-diacyl-3-beta-d-galactosyl-sn-glycerol (n-c18:0), 1,2-diacyl-3-beta-d-galactosyl-sn-glycerol (n-c18:1), 1,2-diacyl-3-beta-d-galactosyl-sn-glycerol (n-c18:2), 1,2-diacyl-3-beta-d-galactosyl-sn-glycerol (n-c18:3) |
| DGaS_140 | Lipid Biosynthesis | UCYN_04040, UCYN_04050, UCYN_05060,UCYN_05260, UCYN_05990, UCYN_08180, UCYN_11640, UCYN_00950, UCYN_01590, UCYN_02810, UCYN_03630, UCYN_03810 | 1 | digalactosyl-diacylglycerol (n-c14) |
| DGaS_180 | Lipid Biosynthesis |  | 1 | digalactosyl-diacylglycerol (n-c18:0) |
| DGaS_161 | Lipid Biosynthesis |  | 1 | digalactosyl-diacylglycerol (n-c16:1) |
| DGaS_160 | Lipid Biosynthesis |  | 1 | digalactosyl-diacylglycerol (n-c16) |
| DGaS_183 | Lipid Biosynthesis |  | 1 | Digalactosyl-diacylglycerol(n-C18:3) |
| DGaS_182 | Lipid Biosynthesis |  | 1 | digalactosyl-diacylglycerol (n-c18:2) |
| DGaS_181 | Lipid Biosynthesis |  | 1 | digalactosyl-diacylglycerol (n-c18:1) |
| GaT_140 | Lipid Biosynthesis |  | 2 | digalactosyl-diacylglycerol (n-c14), 1,2-diacyl-3-beta-d-galactosyl-sn-glycerol (n-c14) |
| GaT_161 | Lipid Biosynthesis |  | 2 | digalactosyl-diacylglycerol (n-c16:1), 1,2-diacyl-3-beta-d-galactosyl-sn-glycerol (n-c16:1) |
| GaT_183 | Lipid Biosynthesis |  | 2 | Digalactosyl-diacylglycerol(n-C18:3), 1,2-diacyl-3-beta-d-galactosyl-sn-glycerol (n-c18:3) |
| GaT_182 | Lipid Biosynthesis |  | 2 | digalactosyl-diacylglycerol (n-c18:2), 1,2-diacyl-3-beta-d-galactosyl-sn-glycerol (n-c18:2) |
| GaT_180 | Lipid Biosynthesis |  | 2 | digalactosyl-diacylglycerol (n-c18:0), 1,2-diacyl-3-beta-d-galactosyl-sn-glycerol (n-c18:0) |
| GaT_160 | Lipid Biosynthesis |  | 2 | digalactosyl-diacylglycerol (n-c16), 1,2-diacyl-3-beta-d-galactosyl-sn-glycerol (n-c16) |
| GaT_181 | Lipid Biosynthesis |  | 2 | digalactosyl-diacylglycerol (n-c18:1), 1,2-diacyl-3-beta-d-galactosyl-sn-glycerol (n-c18:1) |
| rxn09109 | Lipid Biosynthesis | UCYN_02790 | 1 | phosphatidylglycerol ditetradecanoyl |
| rxn09106 | Lipid Biosynthesis | UCYN_03970 | 1 | phosphatidylglycerol dioctadecanoyl |
| rxn09107 | Lipid Biosynthesis | UCYN_03970 | 1 | phosphatidylglycerol dioctadec-11-enoyl |
| rxn09104 | Lipid Biosynthesis | UCYN_03970 | 1 | phosphatidylglycerol dihexadecanoyl |
| rxn09105 | Lipid Biosynthesis | UCYN_03970 | 1 | phosphatidylglycerol dihexadec-9-enoyl |
| rxn09102 | Lipid Biosynthesis | UCYN_03970 | 1 | phosphatidylglycerol ditetradecanoyl |
| rxn09111 | Lipid Biosynthesis | UCYN_02790 | 1 | phosphatidylglycerol dihexadecanoyl |
| rxn09113 | Lipid Biosynthesis | UCYN_02790 | 1 | phosphatidylglycerol dioctadecanoyl |
| rxn09112 | Lipid Biosynthesis | UCYN_02790 | 1 | phosphatidylglycerol dihexadec-9-enoyl |
| rxn09114 | Lipid Biosynthesis | UCYN_02790 | 1 | phosphatidylglycerol dioctadec-11-enoyl |
| SQVT_182 | Lipid Biosynthesis | UCYN_05060 | 1 | sulfoquinovosyldiacylglycerol n-c18:2 |
| SQVT_183 | Lipid Biosynthesis | UCYN_05060 | 1 | sulfoquinovosyldiacylglycerol n-c18:3 |
| SQVT_180 | Lipid Biosynthesis | UCYN_05060 | 1 | sulfoquinovosyldiacylglycerol n-c18:0 |
| SQVT_181 | Lipid Biosynthesis | UCYN_05060 | 1 | sulfoquinovosyldiacylglycerol n-c18:1 |
| rxn08312 | Lipid Biosynthesis | UCYN_01910 | 1 | phosphatidylglycerol dioctadec-11-enoyl |
| SQVT_160 | Lipid Biosynthesis | UCYN_05060 | 1 | sulfoquinovosyldiacylglycerol n-c16:0 |
| SQVT_161 | Lipid Biosynthesis | UCYN_05060 | 1 | sulfoquinovosyldiacylglycerol n-c16:1 |
| rxn08310 | Lipid Biosynthesis | UCYN_01910 | 1 | phosphatidylglycerol dihexadec-9-enoyl |
| rxn08311 | Lipid Biosynthesis | UCYN_01910 | 1 | phosphatidylglycerol dioctadecanoyl |
| rxn08307 | Lipid Biosynthesis | UCYN_01910 | 1 | phosphatidylglycerol ditetradecanoyl |
| rxn08309 | Lipid Biosynthesis | UCYN_01910 | 1 | phosphatidylglycerol dihexadecanoyl |
| SQVT_140 | Lipid Biosynthesis | UCYN_05060 | 1 | sulfoquinovosyldiacylglycerol n-c14:0 |
| G3P_AT182 | Lipid Biosynthesis | UCYN_03550 | 3 | sulfoquinovosyldiacylglycerol n-c18:2, digalactosyl-diacylglycerol (n-c18:2), 1,2-diacyl-3-beta-d-galactosyl-sn-glycerol (n-c18:2) |
| G3P_AT183 | Lipid Biosynthesis | UCYN_03550 | 3 | sulfoquinovosyldiacylglycerol n-c18:3, Digalactosyl-diacylglycerol(n-C18:3), 1,2-diacyl-3-beta-d-galactosyl-sn-glycerol (n-c18:3) |
| rxn09065 | Lipid Biosynthesis | UCYN_04040 | 3 | sulfoquinovosyldiacylglycerol n-c16:0, digalactosyl-diacylglycerol (n-c16), 1,2-diacyl-3-beta-d-galactosyl-sn-glycerol (n-c16) |
| AG3P_OAT183 | Lipid Biosynthesis | UCYN_09400 | 3 | sulfoquinovosyldiacylglycerol n-c18:3, Digalactosyl-diacylglycerol(n-C18:3), 1,2-diacyl-3-beta-d-galactosyl-sn-glycerol (n-c18:3) |
| rxn09063 | Lipid Biosynthesis | UCYN_04040 | 3 | sulfoquinovosyldiacylglycerol n-c14:0, digalactosyl-diacylglycerol (n-c14), 1,2-diacyl-3-beta-d-galactosyl-sn-glycerol (n-c14) |
| rxn09067 | Lipid Biosynthesis | UCYN_04040 | 3 | sulfoquinovosyldiacylglycerol n-c18:0, digalactosyl-diacylglycerol (n-c18:0), 1,2-diacyl-3-beta-d-galactosyl-sn-glycerol (n-c18:0) |
| rxn09066 | Lipid Biosynthesis | UCYN_04040 | 3 | sulfoquinovosyldiacylglycerol n-c16:1, digalactosyl-diacylglycerol (n-c16:1), 1,2-diacyl-3-beta-d-galactosyl-sn-glycerol (n-c16:1) |
| rxn09068 | Lipid Biosynthesis | UCYN_04040 | 3 | sulfoquinovosyldiacylglycerol n-c18:1, digalactosyl-diacylglycerol (n-c18:1), 1,2-diacyl-3-beta-d-galactosyl-sn-glycerol (n-c18:1) |
| AG3P_OAT182 | Lipid Biosynthesis | UCYN_09400 | 3 | sulfoquinovosyldiacylglycerol n-c18:2, digalactosyl-diacylglycerol (n-c18:2), 1,2-diacyl-3-beta-d-galactosyl-sn-glycerol (n-c18:2) |
| P_Phos183 | Lipid Biosynthesis | UCYN_04040 | 3 | sulfoquinovosyldiacylglycerol n-c18:3, Digalactosyl-diacylglycerol(n-C18:3), 1,2-diacyl-3-beta-d-galactosyl-sn-glycerol (n-c18:3) |
| P_Phos182 | Lipid Biosynthesis | UCYN_04040 | 3 | sulfoquinovosyldiacylglycerol n-c18:2, digalactosyl-diacylglycerol (n-c18:2), 1,2-diacyl-3-beta-d-galactosyl-sn-glycerol (n-c18:2) |
| rxn08549 | Lipid Biosynthesis | UCYN_03550 | 4 | sulfoquinovosyldiacylglycerol n-c16:0, phosphatidylglycerol dihexadecanoyl, digalactosyl-diacylglycerol (n-c16),  1,2-diacyl-3-beta-d-galactosyl-sn-glycerol (n-c16) |
| rxn08547 | Lipid Biosynthesis | UCYN_03550 | 4 | sulfoquinovosyldiacylglycerol n-c14:0, phosphatidylglycerol ditetradecanoyl, digalactosyl-diacylglycerol (n-c14),  1,2-diacyl-3-beta-d-galactosyl-sn-glycerol (n-c14) |
| rxn08088_mod | Lipid Biosynthesis | UCYN_09400 | 4 | sulfoquinovosyldiacylglycerol n-c18:0, phosphatidylglycerol dioctadecanoyl, digalactosyl-diacylglycerol (n-c18:0),  1,2-diacyl-3-beta-d-galactosyl-sn-glycerol (n-c18:0) |
| rxn08551_mod | Lipid Biosynthesis | UCYN_03550 | 4 |  |
| rxn08084 | Lipid Biosynthesis | UCYN_09400 | 4 | sulfoquinovosyldiacylglycerol n-c14:0, phosphatidylglycerol ditetradecanoyl, digalactosyl-diacylglycerol (n-c14),  1,2-diacyl-3-beta-d-galactosyl-sn-glycerol (n-c14) |
| rxn08086 | Lipid Biosynthesis | UCYN_09400 | 4 | sulfoquinovosyldiacylglycerol n-c16:0, phosphatidylglycerol dihexadecanoyl, digalactosyl-diacylglycerol (n-c16),   1,2-diacyl-3-beta-d-galactosyl-sn-glycerol (n-c16) |
| rxn08087 | Lipid Biosynthesis | UCYN_09400 | 4 | sulfoquinovosyldiacylglycerol n-c16:1, phosphatidylglycerol dihexadec-9-enoyl, digalactosyl-diacylglycerol (n-c16:1),   1,2-diacyl-3-beta-d-galactosyl-sn-glycerol (n-c16:1) |
| rxn08550 | Lipid Biosynthesis | UCYN_03550 | 4 |  |
| rxn08089 | Lipid Biosynthesis | UCYN_09400 | 4 | sulfoquinovosyldiacylglycerol n-c18:1, phosphatidylglycerol dioctadec-11-enoyl, digalactosyl-diacylglycerol (n-c18:1),  1,2-diacyl-3-beta-d-galactosyl-sn-glycerol (n-c18:1) |
| rxn08552 | Lipid Biosynthesis | UCYN_03550 | 4 |  |
| rxn03159 | Lipopolysaccharide Biosynthesis | UCYN_01220 | 1 | lipid A disaccharide |
| rxn06729 | Lipopolysaccharide Biosynthesis | UCYN_01230 | 1 | lipid A disaccharide |
| UGSAT | Lipopolysaccharide Biosynthesis | UCYN_04090 | 1 | lipid A disaccharide |
| rxn03130 | Lipopolysaccharide Biosynthesis | UCYN_01780, UCYN_05220 | 1 | lipid A disaccharide |
| rxn03146 | Lipopolysaccharide Biosynthesis | UCYN_03720 | 1 | lipid A disaccharide |
| rxn06874 | Nitrogen Metabolism | UCYN_06160, UCYN_06140, UCYN_06150 | 68 | CoA, FAD, s-adenosyl-l-methionine, AMP, acetyl-coa, l-glutamate, udp-glucose, l-glycine, l-alanine, GTP, l-lysine, l-aspartate,  glutathione, CMP, l-arginine, CTP, l-glutamine, l-serine, l-methionine, UTP, l-tryptophan, l-phenylalanine, l-tyrosine, malonyl-coa, succinyl-coa, tetrahydrofolate, UMP, uracil, biotin, l-leucine, dATP, l-histidine, 5,10-methylenetetrahydrofolate, GMP, adenine, l-proline, l-asparagine, l-valine, l-threonine, adenosine, thymidine, 10-formyltetrahydrofolate, guanine, riboflavin, dGTP, inosine, uridine, spermidine, deoxyguanosine, cytosine, guanosine, l-isoleucine, 5-methyltetrahydrofolate, dCTP, TTP, cytidine, l-cystine, deoxyuridine, deoxyadenosine, deoxycytidine, xanthosine, l-selenocysteine, dtdp-rhamnose, cdp-3,6-dideoxy-d-mannose, lipid A disaccharide, chlorophyll a, l-selenomethionine, Heme O |
| rxn01997 | Other | UCYN_11760 | 1 | dtdp-rhamnose |
| rxn02003 | Other | UCYN_12340 | 1 | dtdp-rhamnose |
| rxn02000 | Other | UCYN_11610 | 1 | dtdp-rhamnose |
| rxn05289 | Other | UCYN_01450 | 2 | dGTP, deoxyguanosine |
| rxn01987 | Pentose phosphate pathway | UCYN_09840 | 1 | deoxyribose |
| rxn04705 | Porphyrin and Chlorophyll Metabolism | UCYN_10050 | 1 | chlorophyll a |
| rxn04161 | Porphyrin and Chlorophyll Metabolism | UCYN_08890 | 1 | chlorophyll a |
| rxn04160 | Porphyrin and Chlorophyll Metabolism | UCYN_05200, UCYN_07820, UCYN_07810 | 1 | chlorophyll a |
| rxn00224 | Porphyrin and Chlorophyll Metabolism | UCYN_06750 | 1 | Heme O |
| rxn02959 | Porphyrin and Chlorophyll Metabolism | UCYN_08000 | 1 | chlorophyll a |
| rxn02733 | Porphyrin and Chlorophyll Metabolism | UCYN_00100 | 1 | chlorophyll a |
| rxn02288 | Porphyrin and Chlorophyll Metabolism | UCYN_03370 | 2 | chlorophyll a, Heme O |
| rxn09179 | Porphyrin and Chlorophyll Metabolism | UCYN_10660 | 2 | chlorophyll a, Heme O |
| rxn01218 | Purine and Pyrimidine Metabolism | UCYN_03450 | 1 | deoxycytidine |
| rxn01219 | Purine and Pyrimidine Metabolism | UCYN_03860 | 1 | deoxycytidine |
| rxn01507 | Purine and Pyrimidine Metabolism | UCYN_03450 | 1 | deoxyadenosine |
| rxn01509 | Purine and Pyrimidine Metabolism | UCYN_07300 | 1 | deoxyguanosine |
| rxn00834 | Purine and Pyrimidine Metabolism | UCYN_06390 | 1 | xanthosine |
| rxn01445 | Purine and Pyrimidine Metabolism | UCYN_03450 | 1 | deoxyguanosine |
| rxn00832 | Purine and Pyrimidine Metabolism | UCYN_01280, UCYN_05340 | 1 | xanthosine |
| rxn01353 | Purine and Pyrimidine Metabolism | UCYN_12360 | 1 | dGTP |
| rxn01961 | Purine and Pyrimidine Metabolism | UCYN_03450 | 1 | xanthosine |
| rxn00913 | Purine and Pyrimidine Metabolism | UCYN_03450 | 1 | guanosine |
| rxn00363 | Purine and Pyrimidine Metabolism | UCYN_03450 | 1 | cytidine |
| rxn00708 | Purine and Pyrimidine Metabolism | UCYN_03450 | 1 | uridine |
| rxn01127 | Purine and Pyrimidine Metabolism | UCYN_10460 | 1 | deoxyadenosine |
| rxn00132 | Purine and Pyrimidine Metabolism | UCYN_03450 | 1 | adenosine |
| rxn00237 | Purine and Pyrimidine Metabolism | UCYN_12360 | 2 | GTP, riboflavin |
| rxn05233 | Purine and Pyrimidine Metabolism | UCYN_04220, UCYN_12420 | 2 | dGTP, deoxyguanosine |
| rxn00139 | Purine and Pyrimidine Metabolism | UCYN_04810 | 3 | s-adenosyl-l-methionine, AMP, adenosine |
| rxn00119 | Purine and Pyrimidine Metabolism | UCYN_01750,  UCYN_03860 | 3 | udp-glucose, UTP, lipid A disaccharide |
| rxn00117 | Purine and Pyrimidine Metabolism | UCYN_12360 | 24 | sulfoquinovosyldiacylglycerol n-c14:0, sulfoquinovosyldiacylglycerol n-c16:0, sulfoquinovosyldiacylglycerol n-c16:1,  sulfoquinovosyldiacylglycerol n-c18:0, sulfoquinovosyldiacylglycerol n-c18:1, sulfoquinovosyldiacylglycerol n-c18:2, sulfoquinovosyldiacylglycerol n-c18:3, udp-glucose, UTP, lipid A disaccharide, digalactosyl-diacylglycerol (n-c14), digalactosyl-diacylglycerol (n-c16), digalactosyl-diacylglycerol (n-c16:1), digalactosyl-diacylglycerol (n-c18:0), digalactosyl-diacylglycerol (n-c18:1), digalactosyl-diacylglycerol (n-c18:2), Digalactosyl-diacylglycerol(n-C18:3), 1,2-diacyl-3-beta-d-galactosyl-sn-glycerol (n-c14), 1,2-diacyl-3-beta-d-galactosyl-sn-glycerol (n-c16), 1,2-diacyl-3-beta-d-galactosyl-sn-glycerol (n-c16:1), 1,2-diacyl-3-beta-d-galactosyl-sn-glycerol (n-c18:0), 1,2-diacyl-3-beta-d-galactosyl-sn-glycerol (n-c18:1), 1,2-diacyl-3-beta-d-galactosyl-sn-glycerol (n-c18:2), 1,2-diacyl-3-beta-d-galactosyl-sn-glycerol (n-c18:3) |
| rxn02475 | Riboflavin Metabolism | UCYN_02660 | 2 | FAD, riboflavin |
| rxn02474 | Riboflavin Metabolism | UCYN_11420 | 2 | FAD, riboflavin |
| rxn03080 | Riboflavin Metabolism | UCYN_01730 | 2 | FAD, riboflavin |
| rxn05040 | Riboflavin Metabolism | UCYN_01560 | 2 | FAD, riboflavin |
| rxn00048 | Riboflavin Metabolism | UCYN_02700 | 2 | FAD, riboflavin |
| rxn00300 | Riboflavin Metabolism | UCYN_01560 | 2 | FAD, riboflavin |
| rxn05039 | Riboflavin Metabolism | UCYN_03450 | 2 | FAD, riboflavin |
| rxn09399 | Starch and Sucrose Metabolism | UCYN_00450 | 1 | glycogen |
| rxn00695 | Starch and Sucrose Metabolism | UCYN_08010 | 1 | glycogen |
| rxn13643 | Starch and Sucrose Metabolism | UCYN_08480 | 1 | glycogen |
| rxn03843 | Terpenoid Backbone Biosynthesis | UCYN_03140 | 1 | di-trans poly-cis-undecaprenyl diphosphate |
| rxn04308 | Terpenoid Backbone Biosynthesis | UCYN_03140 | 1 | di-trans poly-cis-undecaprenyl diphosphate |
| rxn01487 | Terpenoid Backbone Biosynthesis | UCYN_09570 | 1 | chlorophyll a |
| rxn03909 | Terpenoid Backbone Biosynthesis | UCYN_03050 | 2 | di-trans poly-cis-undecaprenyl diphosphate, chlorophyll a |
| rxn01486 | Terpenoid Backbone Biosynthesis | UCYN_00870 | 2 | chlorophyll a, all-trans-zeta-carotene |
| MECDPDH | Terpenoid Backbone Biosynthesis | UCYN_06810 | 3 | di-trans poly-cis-undecaprenyl diphosphate, chlorophyll a, all-trans-zeta-carotene |
| rxn03907 | Terpenoid Backbone Biosynthesis | UCYN_05070 | 3 | di-trans poly-cis-undecaprenyl diphosphate, chlorophyll a, all-trans-zeta-carotene |
| rxn03908 | Terpenoid Backbone Biosynthesis | UCYN_08710 | 3 | di-trans poly-cis-undecaprenyl diphosphate, chlorophyll a, all-trans-zeta-carotene |
| rxn03910 | Terpenoid Backbone Biosynthesis | UCYN_05070 | 3 | di-trans poly-cis-undecaprenyl diphosphate, chlorophyll a, all-trans-zeta-carotene |
| rxn03958 | Terpenoid Backbone Biosynthesis | UCYN_00200 | 3 | di-trans poly-cis-undecaprenyl diphosphate, chlorophyll a, all-trans-zeta-carotene |
| rxn00438 | Thiamine Metabolism | UCYN_03940 | 1 | thiamin diphosphate |

**References**

[1] A. W. Thompson *et al.*, “Unicellular cyanobacterium symbiotic with a single-celled eukaryotic alga,” *Science (80-. ).*, 2012, doi: 10.1126/science.1222700.

[2] H. Kacser, J. A. Burns, H. Kacser, and D. A. Fell, “The control of flux: 21 years on,” *Biochem. Soc. Trans.*, 1995, doi: 10.1042/bst0230341.

[3] R. Moreno-Sánchez, E. Saavedra, S. Rodríguez-Enríquez, and V. Olín-Sandoval, “Metabolic Control Analysis: A tool for designing strategies to manipulate metabolic pathways,” *Journal of Biomedicine and Biotechnology*. pp. 1–30, 2008, doi: 10.1155/2008/597913.
