# Supplementary material for: Elucidation of trophic interactions in an unusual single-cell nitrogen-fixing symbiosis using metabolic modeling
Source: PLoS Comput Biol. 2021 May 7;17(5):e1008983. doi: 10.1371/journal.pcbi.1008983 (PMC8143392; doi:10.1371/journal.pcbi.1008983)
Supplement: Attachment — Submitted filename: ReviewReplies_020821.docx [file pcbi.1008983.s006.docx]

**Comments to the Authors**

Reviewer #1: I have read the manuscript entitled "Elucidation of trophic interactions in an unusual single-cell nitrogen-fixing symbiosis using metabolic modeling" by Sarkar et al. submitted for publication to Plos Computational Biology. The manuscript deals with the metabolic reconstruction and modeling of the trophic interactions in a single-cell nitrogen fixing symbiosis, the one established by UCYN-A and a halophyte algal host.

In general, it feels like the reconstruction was properly performed (despite a lack of details on the actual implementation of the model, see below) but, on the other end, other additional aspects could have been investigated about this interesting biological system using the integrated model.

This system is characterized by a tremendous lack of information, mainly due to the fact that by both UCYN-A and its host are uncultured. The work done by the authors is profoundly influenced by this as both the reconstructions were built using other (more or less) related organisms as template.

In this context, it seems that the authors made all the efforts to include the available annotations (C. Parka and B. Bigelowii are said to be "partially sequenced". Btw, what does this mean? Does it mean that only a small fraction of the genome is actually sequenced or that the whole sequence is available as draft?) of the host and the symbiont to reconstruct a model that is as close as possible to the actual metabolic network of the two. It would be interesting to know more details about the two reconstructions and a possible guess on how complete they actually are. For example, how many genes of the two organisms were actually included in the corresponding reconstructions? How many transport reactions were included in the reconstructions? A few more tables including this information would facilitate the reading.

>> For both *C. parkeae* and *B. bigelowii*, only segments of their ribosomal DNA have been sequenced till date to enable phylogenetic studies (Takano et al., Mar Micropaleontology, 2006; Hagino et al., Mar Micropaleontology, 2009; Thompson et al., Science, 2012). This precludes including any information from known UCYN hosts in the GSM reconstruction, and was the primary motivation behind using *C. tobin* as the representative host. This has now been clarified in the manuscript (L408-410).

I am deeply concerned by the fact that the authors did not provide (as additional material or as link to a public repository) the models and the codes they used to run the simulations. They only provided the models in xls format which is not the standard in genome scale metabolic modeling. I was expecting sbml files or something comparable. The description of the implementation of the model is restricted to lines 459-464 which do not provide enough details/material to reproduce the results or apply the proposed approach to other study-cases.

>> We were planning to make this information after the manuscript was reviewed. In response to the reviewer’s request we have provided SBML (v3) files for both GSMs and GAMS scripts for performing all analysis reported in the manuscript (https://github.com/maranasgroup/UCYN-A-symbiosis-metabolic-modeling).

The authors have used constrained-based metabolic modeling to infer the interconnection between these two organisms and the minimal set of nutrients that are probably exchanged by the two organisms to reciprocally sustain life. In this "do ut des" system (and in many other symbioses), trade-offs play an interesting role. In this case, the host needs to invest resources to have usable nitrogen back from the symbiont and viceversa. But the question is, which is the limit of the system? Is there a point behind which the system collapses because too many (or too few) resources have been invested by the host (or by the symbiont)? This aspect has been overlooked by the authors but something in the range of a Pareto frontier analysis could highlight interesting features of the system.

>> Dissolved nitrogen in oceanic waters needs to be converted to ammonium in order to support carbon dioxide fixation, and biological nitrogen fixation is known to be limiting throughout large areas of the surface ocean (see Zehr and Capone, Science, 2020 for a detailed review). Thus, the symbiosis between UCYN-A and its host is believed to be obligatory, especially due to lack of free-living observations of either partner (Massana et al., ISME J, 2016), strong coupling between nitrogen and carbon transfer (Krupke et al., ISME J, 2014), and recent observations on low exogenous nitrogen uptake even in a dissolved nitrogen-replete environment (Mills et al., ISME J., 2020). As the biochemical composition of UCYN-A is still uncharacterized, a pareto front analysis based on metabolite sharing and growth rates of the two organisms (as a proxy of organism ‘fitness’) is prohibitive. Thus, we determined the minimal metabolite set that must be exchanged between the two partners so as to enable UCYN-A growth (Figure 1 in the manuscript, section ‘Metabolites transferred from the host to the symbiont’). This can be considered to be the lower bound for metabolite sharing in the symbiosis, below which the system will collapse. This clarification has been added in the revised manuscript (L157-161).

Additionally, the effect of a number of external perturbations on the system could have been explored. I am listing here a few of them but the list can surely be expanded by the authors.

1. What would happen if the host could retrieve fixed nitrogen from the external environment? Would this be possible in a real case scenario? How would this impact the metabolic cross-talk of the two organisms?

>> The distribution and abundance of diazotrophs, N_2_-fixation rates, and foodweb-associated fates of fixed N_2_ in surface oceans remains poorly characterized and an open area of investigation (Zehr and Capone, Science, 2020). Based on prevalence and occurrence data alone, it is plausible that the host can retrieve a fraction of its nitrogen budget from the environment in areas where the UCYN-A symbiosis and diazotrophs such as *Trichodesmium* are found together. A recent study found that the haptophyte host does not assimilate nitrate and meets little of its nitrogen demands via ammonium uptake, even in dissolved inorganic nitrogen replete areas (Mills et al., ISME J., 2020).

In order to assess the impact of fixed nitrogen availability on the host-UCYN-A symbiosis, we varied the host nitrogen uptake from zero (i.e., relying solely on UCYN-A) to the amount needed for maximal growth (i.e., all nitrogen requirements being satisfied from the environment). Carbon and nitrogen sharing between the two partners was also allowed to vary from the experimentally observed ratios. The resultant maximal host biomass production flux and UCYN-A N_2_ fixation flux was then calculated for each case (see Figure 1 below). As expected, the host biomass increases as the nitrogen supply is increased (either from UCYN-A or the environment), but also competes with carbon transfer to UCYN-A. In the limiting case when UCYN-A is the sole source of fixed nitrogen, increasing carbon transfer translates to a higher nitrogen fixation flux (due to increased availability of reducing equivalents) but only to the extent wherein it does not compete with carbon sequestration for biomass production. At the opposite end of the spectrum, where all nitrogen requirements are fulfilled by the environment, increasing carbon transfer leads to a decrease in host biomass production. This transition from nitrogen-limited to carbon-limited growth thus dictates the limits of this symbiotic system. This analysis has been included in Supplementary Material.

**Figure 1: Predicted algal host growth rate with variations in metabolite transfer and host nitrogen uptake.** Nitrogen transfer (from UCYN-A to host, y-axis) and carbon transfer (from host to UCYN-A, x-axis) were varied, and the maximal host biomass production flux calculated (in hr^-1^ , see color bar on bottom right). Nitrogen uptake for the host was also varied, ranging from zero (denoting no nitrogen supplementation from the environment and relying solely on UCYN-A) to 100% (denoting all nitrogen requirements being satisfied from the environment) (see individual plot titles).

2. There is no investigation on the role of specific genes in the establishment and maintenance of the symbiosis. A gene-essentially analysis (for example in different environmental conditions, as suggested above) might have revealed interesting insights.

>> We believe that gene essentiality is poorly defined in the current context, given the uncertainty in host metabolic capabilities (due to lack of sequenced genomes for the natural hosts), absence of a UCYN-A biomass composition, and UCYN-A’s metabolic deficiencies potentially complemented by metabolite transport from the host. However, following the reviewer’s suggestion we performed an essentiality analysis by systematically deleting UCYN-A model reactions and determining the subsequent maximal production flux for each biomass precursor. Our analysis indicates that 154 reactions (or 25.4%) are essential in UCYN-A, which translates to 155 essential metabolic genes (or 38.8%). This fraction is similar to other minimal cyanobacteria (such as *P. marinus* with 47% essential genes (Casey et al., mSys, 2016)), reinforcing the streamlined metabolism from adaptive gene loss hypothesis (Bombar et al., ISME Jour., 2014). The manuscript has been updated to reflect this analysis (L428-429), and the list of all essential genes and the corresponding blocked biomass precursor(s) are listed in Supplementary Material.

3. A similar analysis could be performed at the reaction-level, for example constraining the flux in all the reactions of the model (one at the time, obviously) to higher or lower values in respect to the reference FBA flux distribution.

>> We performed a metabolic control analysis to analyze the effect of reaction-level flux perturbations in UCYN-A on the maximal N_2_ fixation and host biomass production flux. Briefly, host biomass production was first maximized, and the maximal N_2_ fixation flux calculated at that value, followed by parsimonious flux balance analysis to determine the reference flux distribution. Flux through every UCYN-A metabolic reaction was then perturbed (by increasing and decreasing by 10% of the reference value), and the resultant host biomass production and UCYN-A N_2_ fixation determined. As expected, reactions involved in producing reductants NAD(P)H and ATP had a high impact on both host biomass and N_2_ fixation (such as PSI, uptake hydrogenase, and cyclic electron flow). Interestingly, reactions involved in riboflavin metabolism and GTP synthesis (which serves as the precursor for riboflavin) also affected host biomass production. Results from this analysis have now been incorporated in the manuscript (L245-248), and the detailed set included in Supplementary Material.

4. A deeper analysis of the combined effects of CO2 and light on the N2-fixing symbiont should be performed. Is nitrogen fixation influenced by light intensity? Constraining light adsorption at different values and checking what happens I the symbiont model could reveal interesting insights.

>> Constraining light adsorption at different intensities and computing the corresponding host biomass and UCYN-A nitrogen fixation flux yields expected saturating trends (see Figure 2 and 3 below). The symbiotic system is unable to fix nitrogen or produce host biomass when no photons are supplied to the host, due to the lack of fixed carbon. However, UCYN-A photon uptake is inessential as its diazotrophy can be supported by substrate-level phosphorylation from fixed carbon imported from the host.

**Figure 2: Variation of UCYN-A diazotrophy with photon uptake.** The x-axis depicts the photon uptake flux (in mmol gDW^-1^ hr^-1^) and the y-axis depicts the corresponding UCYN-A nitrogen fixation flux (in mmol gDW^-1^ hr^-1^).

**Figure 3: Variation of the host growth rate with photon uptake.** The x-axis depicts the photon uptake flux (in mmol gDW^-1^ hr^-1^) and the y-axis depicts the corresponding host biomass production flux (in hr^-1^).

5. To put the work in perspective the results obtained with this system could be discussed in relation to other similar (but unrelated) symbioses (e.g. the one between plant and bacteria, Medicago and Sinorhizobium).

>> Nitrogen-fixing symbiotic associations are abundant in nature, starting from agricultural to aquatic ecosystems. Such interactions between plants and associative nitrogen-fixing bacteria comprise a number of bacterial genus, such as *Azospirillum*, and cyanobacteria, and plants such as wheat, maize, and rice. These associations range from free-living diazotrophs, to intercellular endophytic associations, and endosymbioses. The underlying molecular mechanisms are equally diverse, with legumes forming nodules to host rhizobium and filamentous cyanobacteria developing specialized cells (called heterocysts) to allow spatial separation of oxygen-sensitive nitrogen fixation and oxygen-evolving photosynthesis. Our results indicate that the priming of nitrogen fixation in the UCYN-A and haptophytic host symbiosis is similar to that seen in heterocysts (as evidenced by the transfer of fixed nitrogen as ammonia or glutamine), but the vastly different metabolic abilities of the two partner and lack of specialized cellular substructures prevents simply classifying UCYN-A as a heterocyst. The streamlined UCYN-A genome suggests that the symbiosis is obligatory, with striking similarities to the algal chloroplast genomes. For example, UCYN-A exists in the cell in two different forms caused by chromosomal rearrangements bracketed by inverted sequence repeat regions. This suggests that this unicellular cyanobacterium is the closest observed instance of a specialized nitrogen-fixing organelle, or ‘nitroplast’. A similar example can be found in the ‘spheroid bodies’ observed in diatoms from the family *Rhopalodiaceae* – these lack genes for both PSI and PSII, and have an incomplete TCA cycle, but possess complete biosynthetic pathways for amino acids, nucleotides, and cofactor in a genome that is almost twice the size of UCYN-A (1.44 Mbp vs 2.79 Mbp) (Nakayama et al., PNAS, 2014).

Thus, despite the strong coevolutionary histories observed in a majority of symbioses in nature, only a few exhibit the loss of individual autonomies. Herein lies the distinction that singles out the UCYN-A and haptophytic host unicellular association and enables us to study the evolutionary transition between symbiotic partnerships and new, integrated organisms. Parts of this explanation has been appended in the revised manuscript in the ‘Introduction’.

Reviewer #2: Overall, this is an interesting paper that explores potential trophic interactions between two uncultured species, with a symbiotic nitrogen fixation and carbon fixation relationship. Although there really aren’t many new computational methods in this paper, it does a nice job of showing how community models can be used to explore these trophic interactions, and the carbon transfer/nitrogen transfer constraints add some novelty, as does the exploration of limits for different O2 scavenging processes.

I have only a few minor comments and suggestions for this paper:

Comments:

1.) The description of the biomass in the results seems to imply all of the coefficients are equal and minimal in the UCYN-A model:

“FBA was carried out by requiring that each UCYN-A biomass precursor was produced at a minimal level (as the exact biomass composition is unknown)”

But the methods say the biomass composition from C. reinhardtii was used. Perhaps the sentence in the results could be clarified?

>> Biomass composition from *C. reinhardtii* was used for the host GSM, and UCYN-A’s biomass precursors were adapted from *Cyanothece* sp. ATCC 51142 and *P. marinus*. We have clarified this in the manuscript (L140-142).

2.) When enforcing boolean matching of the GPR during reconstruction of UCYN-A, how many partial matches were rejected? Did gap filling add reactions associated with partial matches back into the model? Could it be possible UCYN-A has some alternative protein complex stoichiometry?

>> It is plausible that given UCYN-A’s streamlined genome, enzymatic functions and protein complex stoichiometry have similarly evolved. However, we do not deem that as being probable as the commonly observed evolutionary modifications include genome reduction by gene loss (as seen in *P. marinus* by the absence of phycoerythrin and nitrate/nitrite reductases (García-Fernández et al., Microbiol and Mol Bio Rev, 2004)). Furthermore, a bidirectional BLAST was performed during UCYN-A GSM reconstruction with an e-value cutoff of 10^-5^, preventing the inclusion of partial matches.

During gap filling, a protein BLAST was first performed to confirm the presence of a metabolic function and only then was a reaction added to the network. As metabolic deficiencies are expected to be complemented by the host, reaction addition during gapfilling was subjected to strict scrutiny, with a preference given to adding reactions to the host metabolic model. Model statistics such as number of metabolites, reactions, genes, and gap filling have been included in the Supplementary Material.

3.) It seems like some model stats in the results would be helpful. The number of reactions. The number of genes. The amount of gap filling required.

>> Model statistics such as number of metabolites, reactions, genes, and gapfilling have been included in Supplementary Material.

4.) The authors make a significant point that UCYN-A is not cultured, yet they do have a genome for annotation and reconstruction. It seems like they should describe the provenance of this genome. Citations are made, but some minimal information on this in the paper itself (a few sentences) would be helpful. Is the genome closed? Given it’s minimized nature, errors in assembly or binning could be significant.

>> The UCYN-A genome sequence (as reported in Tripp et al., Nature, 2010) was first assembled into one scaffold containing gaps of known lengths, and then closed using a combination of contig pooling and PCR. We believe that commenting on the genome assembly is outside the purview of this manuscript, but have included the above information in the revised draft (L389-391).

5.) It’s not totally clear from the results. Does UCYN-A have all the enzyme components for both oxygen scavenging system explored?

>> UCYN-A has the necessary genes for cytochrome c oxidase (reaction ID CYO1b2pp_syn, gene-protein-reaction relationship (UCYN_12280 and UCYN_12290 and UCYN_12300) and (UCYN_02310)) and Mehler reaction (reaction ID R_MEHLER, gene UCYN_04350). This has now been clarified in the revised manuscript (L297-299).

6.) It seems like the oxygen scavenging comparison should include the costs of dealing with the H2O2 byproduct of the Mehler reaction. It’s mentioned in the text that systems exist to deal with this byproduct, but what are the metabolic costs?

>> The peroxide reducing enzymes superoxide dismutase (*sod1*) and peroxiredoxins (*prxR*) do not incur additional metabolic costs in terms of ATP or reducing equivalents NAD(P)H input.

7.) Perhaps in figure 4 the distinctive reactions in each mechanism could be highlighted with a different color to help clarify this to the reader?

>> We thank the reviewer for this comment and Figure 4 has been updated to accommodate the suggested change.

Note to reviewers: Previously, we used an ‘unpublished data’ reference to denote the partially-sequenced nature of host genomes. This has now been replaced with references (Thompson et al., 2012, Science), (Takano et al., 2006, Mar. Micropaleontol.), and (Hagino et al., 2009, Mar. Micropaleontol.) summarizing the current status of host sequencing (L408-409).
